# Permanent Unilateral Hearing Loss (UHL) and Childhood Development

**DOI:** 10.1007/s40136-018-0185-5

**Published:** 2018-02-15

**Authors:** Judith E. C. Lieu

**Affiliations:** Department of Otolaryngology-Head and Neck Surgery, Washington University School of Medicine, 660 S. Euclid Ave., Campus Box 8115, St. Louis, MO 63110 USA

**Keywords:** Unilateral hearing loss, Children, Speech and language, Cognition, Executive functions, Quality of life

## Abstract

**Purpose of Review:**

The aim of this study is to summarize the consequences of permanent unilateral hearing loss (UHL) on the development of children as documented in the recent literature.

**Recent Findings:**

Congenital and early-identified UHL places young children at risk for delays in speech-language development. School-aged children with UHL score lower on standardized tests of language and cognition and need increased assistance in school for educational and behavioral issues than siblings with normal hearing, and report lower hearing-related quality of life, similar to children with bilateral hearing loss (HL). Early intervention, including use of hearing amplification devices, might ameliorate some of those affects. For a child with mild to severe UHL at presentation, the risk of progression of HL in the worse-hearing ear may be as high as 40%, and the risk of progression to bilateral HL approaches 20%.

**Summary:**

Although UHL can adversely affect the development of children, how to mitigate those effects requires investigation.

## Introduction

Although studies in the 1980s suggested that school-aged children with unilateral hearing loss (UHL) were at risk for adverse educational outcomes [1–3] the advent of widespread newborn hearing screening identified infants and toddlers with congenital UHL routinely for the first time. The influx of newly early-identified young children with UHL brought with them the chagrin among hearing professionals stemming from the scant evidence from which to base recommendations for management of these children. The National Workshop on Mild and Unilateral Hearing Loss was convened in July 2005 by the Centers for Disease Control and Prevention (CDC), Early Hearing Detection and Intervention Program to provide recommendations and plans to fill gaps in the evidence [4]. Many investigators have risen to the challenge of filling that void of data regarding the consequences of UHL. Other investigators have evaluated the role of intervention through amplification devices. Thus, the objective of this review is to summarize the evidence that UHL affects the development of children from multiple aspects and to suggest possible management strategies to mitigate some of these developmental consequences.

## Prevalence of UHL

The CDC workshop recommended the following definition of UHL: “A permanent unilateral hearing loss exists when the diagnosis indicates there is a calculated or predicted average pure tone air conduction threshold at 0.5, 1, 2 kHz of any level greater than or equal to 20 dB HL or pure tone air conduction thresholds greater than 25 dB HL at two or more frequencies above 2 kHz in the affected ear with an average pure tone air conduction threshold in the good ear less than or equal to 15 dB” [4]. Despite this recommendation, studies that estimate the prevalence of UHL have used other definitions, varied methods for ascertaining hearing thresholds, and sampled populations that do not represent all children at birth or later in life [5]. Thus, estimates from newborn hearing screening program suggest approximately one congenital UHL per 1000 births, with UHL thought to comprise about one third of all children born with hearing loss [6, 7]. The prevalence of UHL increases with age as delayed-onset congenital hearing loss (HL) and acquired etiologies emerge, such that the prevalence increases to 14% among adolescents ages 12–19 years [8]. Ross et al. demonstrated that using variable case definition results in estimates of prevalence between 3.0 and 6.3% among children 6–19 years of age [9].

## Progression of UHL

Several studies have followed children with UHL to determine rates of progression of UHL in the worse hearing ear and in the normal-hearing (NH) ear (i.e. to bilateral HL). In one region of Canada, Fitzpatrick et al. followed 108 children with UHL identified via newborn hearing screening [10]. Forty-two percent [11] showed deterioration in hearing, and 16 (17%) of them developed bilateral HL. However, only 18 children (17%) presented with profound UHL, which provided a large percentage of children who could possibly lose further hearing. Purcell et al. compared 128 children with UHL for their risk for progression of HL based on the presence of cochlear nerve bony stenosis or cochlear nerve hypoplasia [12]. After a minimum 6 months follow-up, they found that the rates of progressive HL at 12 months were 12% without temporal bone anomaly, 13% with any temporal bone anomaly, and 17% in those specifically with cochlear nerve bony stenosis (HR 2.17, [95% CI 1.01–4.66]). A total of 42 children (33%) demonstrated some progression over time. Because the PTA at the onset of the study period averaged 70 dB, it is likely that many children began with severe-to-profound UHL initially. Paul et al. in France found that among 80 children with UHL, 15 (19%) had progressive UHL, 7 (9%) had fluctuating UHL, and 6 (7.5%) developed bilateral HL. However, 54 (68%) were identified initially as having severe-to-profound UHL [13].

It should be noted that severity of HL cannot be counted as progressing when the children present with profound UHL. Thus, the maximum percentage of progression depends on the percentage who do not present with profound UHL. For instance, Van Beeck Calkoen et al. reviewed 102 children with UHL and noted that 62 (64%) had profound UHL at presentation, with an addition 14 (14%) with severe UHL [14]. Thus, in this group, only 40 children had the possibility for significant progression of UHL thresholds. Risk for progression also varies by etiology, such that children with enlarged vestibular aqueduct (EVA), cochlear canal bony stenosis (or hypoplastic cochlear nerve), and congenital CMV are considered to have a higher risk for progression [12, 15, 16].

## Speech-Language Consequences

### Infant-Toddler and Preschool Speech-Language Development

In the past 5 years, speech-language evaluations of young children with UHL identified before preschool using standard assessments have emerged. Kishon-Rabin et al. assessed auditory behavior and preverbal vocalizations using two parent questionnaires among 34 infants with UHL (median age 9.4 months) compared with 331 infants with NH (median age 9.0 months) [17••]. They noted that auditory behavior as measured by the Infant-Toddler Meaningful Auditory Integration Scale (IT-MAIS) was delayed in 21% of children with UHL compared to 4% in children with NH (OR 3.86), and preverbal vocalization as measured by the Production of Infants Scale Evaluation (PRISE) was delayed in 41% of children with UHL compared to 3.6% of children with NH (OR 8.64).

In a prospective cohort of children identified with HL through Rhode Island newborn hearing screening program, Vohr and colleagues evaluated children at a mean age of 60 months (± 5 months) for language and adaptive behavior using the Reynell Developmental Language Scales and the Vineland Adaptive Behavior Scales [18]. The ten children with mild bilateral HL or UHL were noted to have significantly lower communication, motor skills, and adaptive behavior scores compared to the 74 children with normal hearing. In addition, the children with mild bilateral HL or UHL had similar comprehension and expressive language scores as 19 children with moderate-to-profound bilateral HL.

Fitzpatrick and colleagues evaluated communication development in early-identified children (defined as < 3 years old) with mild bilateral HL and UHL (median age ID = 4.2 months) [19]. This group of children had been assessed at least once at 12, 24, 36, and 48 months old on a battery of speech-language and communication tests including the Parent’s Evaluation of Aural/Oral of Children (PEACH), Early Listening Function (ELF), Children’s Home Inventory for Listening Difficulties (CHILD), MacArthur-Bates Communicative Development Inventory (MCDI), and the Children Development Inventory (CDI). The children were compared in groups of 24 with mild bilateral HL, 31 with UHL, and 45 with NH, with cross-sectional comparisons at each year of age. Most of the children (44 of 55, 80%) had been recommended for amplification, and all had been referred to early intervention services for assessment and early communication development support. They found that only CHILD scores were worse for children with UHL and mild bilateral HL at ages 3 and 4 years. Thus, the investigators concluded that up to age 4 years, children with UHL and mild bilateral HL developed language similarly to their peers with HL. However, a close look at the cross-sectional data shows some large, albeit statistically insignificant differences at age 3 years; the mean CDI Expressive Language quotient was 22 points lower in children with UHL/mild bilateral HL (101.7) compared to NH (123.7), and the MCDI mean length of utterance was 2.4 lower for UHL/mild bilateral HL (8.3) compared to NH (10.7).

### Child and Adolescent Speech-Language Development

A systematic review was published in 2017 to summarize the quantifiable extent of the impact of UHL on children on objective measures of speech and language [20]. Thirteen studies were included in the qualitative synthesis of data. The outcome measures used by the studies were too heterogeneous to complete a quantitative meta-analysis. However, a review of the studies did reveal different categories of outcome. Among the seven studies that showed an overall detrimental effect of UHL on speech and language development, increasingly harmful effects were noted with more severe UHL thresholds. Of the four studies that did not report negative effects on speech and language from UHL, two evaluated children had only mild UHL. In the two studies that examined children with UHL longitudinally, the speech and language deficiencies appeared to improve or resolve, but the studies have been limited by small sample sizes and need for confirmation in larger studies.

**Fig. 1:**
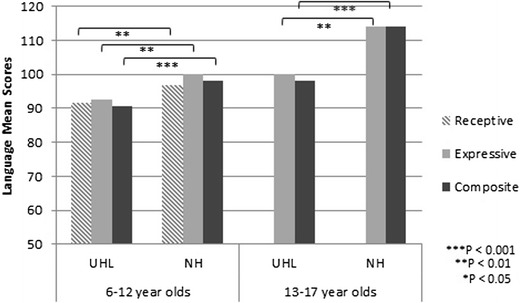
Differences in standardized language scores from childhood to adolescence. Children with unilateral hearing loss (UHL) are compared to their siblings with normal hearing (NH). The effect sizes of the difference in scores appear to enlarge, from 0.5–0.6 SD in elementary school (moderate effect) to 1.0 SD in adolescence (large effect)

In the study by Fischer and Lieu, 20 adolescents (age 12–17 years old) with UHL were compared to 13 adolescent siblings with NH on standardized language tests (Oral and Written Language Scales [OWLS] and the Clinical Evaluation of Language Fundamentals [CELF]) [21]. Adolescents with UHL had worse overall and expressive language scores than controls (98 for UHL vs. 114 for NH, *P* = 0.001; 100 for UHL vs. 114 for NH, *P* = 0.006, respectively). These differences constitute large effect sizes, being 14–16 points or essentially a full SD below their NH sibling controls. These adolescents had previously participated in a similar study as children in elementary school and had smaller differences in scores between the group with UHL as compared to NH (overall scores 90.6 for UHL vs. 98 for NH, *p* < 0.001; expressive scores 92.7 for UHL vs. 100.1 for NH, *p* = 0.003) [22]. Figure 1 shows the relative change in scores over time from the earlier study. Although the language scores for the children with UHL had improved with time, the magnitude of the deficit behind their NH siblings appeared to increase.

A case-control study by Sangen and colleagues in Belgium tested language & auditory development in 21 children 5–15 years old with congenital sensorineural UHL with PTA > 70 dB HL compared to 42 with NH [23]. All of the controls were age and sex-matched. The children with UHL had lower scores on morphology, syntax, and vocabulary as measured by the Clinical Evaluation of Language Fundamentals (CELF-4-NL) and Expressive One Word Picture Vocabulary Test (EOWPVT). In addition, assessment of detailed morphology subskills showed difficulty with verb participles and pronouns in the children with UHL. They also had lower scores on all three subscales of the Speech Spatial and Qualities of Hearing Questionnaire (SSQ). However, they had similar performance on short-term and working memory as measured by the Number Repetition subtest of the CELF-4-NL compared to the children with NH.

## Behavior and Educational Consequences

In a longitudinal study of 46 children with UHL, Lieu et al. documented the parent-reported behavioral problems using the Child Behavior Checklist (CBCL) and educational concerns and issues reported in school records and teacher narratives [24••]. More than 20% of the UHL cohort scored ≤ 3rd percentile for each of the Competency scales (Activities, Social, School, or Total) on the CBCL in the final year, which did not change from the first year. Affective problems declined from 15 to 2% (*p* = 0.02), with possible trends toward lesser attention deficit hyperactive disorder (20 to 5%, *p* = 0.08) and oppositional defiance problems (17 to 7%, *p* = 0.06). However, there were no changes in rates of academic difficulty; teachers reported that 24% had academic area of weakness or executive function on reviews of school-grade report, and approximately 50% continued to have Individualized Educational Plans (IEPs) throughout the 3 years. Although hearing impairment was noted in the IEPs in up to 54%, other reasons for the IEPs were more common.

A study by Reed et al. compared 94 children with right-sided aural atresia, 46 with left-sided aural atresia, and 12 with sensorineural UHL on rates of behavioral and educational problems [25]. They found no significant differences in rates of grade retention (2, 6, and 25% for right, left, and sensorineural UHL, respectively), speech therapy (49, 39, and 42%, for right, left, and sensorineural UHL, respectively), IEPs (36, 37, and 42% for right, left, and sensorineural UHL, respectively), or diagnosis of attention deficit hyperactive disorder (5, 4, and 25%, for right, left, and sensorineural UHL, respectively). However, they observed high rates of special education (11 vs. 26%, *p* = 0.026) and behavioral problems (7 vs. 15%, *p* < 0.001) among children with left aural atresia. Although they did not evaluate this statistically, the rates of special education and behavioral problems among the children with sensorineural UHL were similar or higher than those with left aural atresia.

### Cognitive Development

**Fig. 2:**
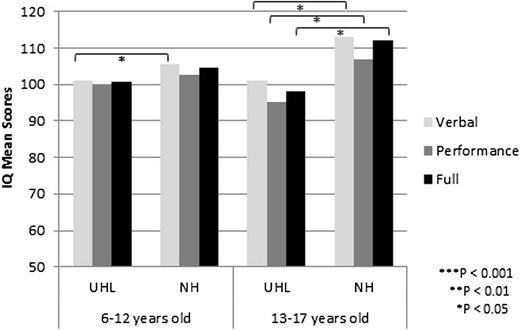
Differences in standardized intelligence quotient scores from childhood to adolescents. Children with UHL are compared to their siblings with NH. The effect sizes of the differences in scores appear to enlarge from 0.2–0.3 SD in elementary school (small effect) to 0.8–0.9 SD in adolescents (large effect)

In addition to testing language skills as reported earlier, Fischer and Lieu assessed cognitive skills using the Wechsler’s Abbreviated Scales of Intelligence (WASI) to assess full-scale, verbal, and performance intelligence quota (IQ) in 20 adolescents (age 12–17 years old) with UHL as compared to 13 adolescent siblings with NH. The adolescents with UHL had lower full-scale (98 vs. 112, *P* = 0.017), verbal (101 vs. 113, *P* = 0.032), and performance IQ (95 vs. 107, *P* = 0.037) compared to their NH-hearing siblings. These 12–14 point decrements are considered large effect size differences in IQ, which had increased in magnitude since elementary age testing [22], as shown in Fig. 2. The differences in IQ scores were further supported in a systematic review by Purcell et al. in which they performed a meta-analysis of IQ scores to compare 6–18 year-old children with UHL (*n* = 173) to NH (*n* = 202) [26]. They noted that children with UHL had full IQ that was − 6.3 (95% CI − 9.1, − 3.5), performance IQ − 3.8 (95% CI − 7.3, − 0.2), and verbal IQ − 4.0 (95% CI − 7.5, − 0.4) compared to children with NH. The effect sizes are 0.42 (moderate) for full IQ and 0.25 to 0.27 (small) for performance and verbal IQ, respectively.

In order to identify and interpret the possible cognitive deficits stemming from UHL, Ead et al. undertook a pilot study of 14 children, age 9–14 years, with severe-to-profound UHL compared with 7 with NH on measures of working memory, processing speed, attention, and phonological processing [27]. They found that phonological processing composite scores were significantly lower for the children with UHL, as well as on a complex letter span task. The latter result suggested impaired executive control function during a verbal task when distracted by irrelevant verbal information.

Other studies have used brain imaging techniques to evaluate the differences in cognitive function between children with UHL and NH. Diffusion tensor imaging (DTI) is a magnetic resonance imaging (MRI) modality that investigates white matter integrity in the brain. Because white matter is comprised of the neuronal tracts that convey the auditory impulses from the cochlea to the brainstem and then auditory cortex and elsewhere, their integrity is essential to efficient distribution of the auditory stimulus. A study done by Rachakonda et al. comparing children with UHL to NH siblings showed that several white matter areas, including both auditory and non-auditory regions of interest, had significantly worse integrity in the children with UHL [28]. These areas included subcortical white matter in Heschl’s gyrus, lateral lemniscus, putamen, anterior limb of the internal capsule, and centrum semiovale. In addition, there were regions of white matter integrity that were significantly associated with clinical outcomes of receiving speech therapy and IEPs and negatively associated with UHL. These included middle cingulate gyrus, middle cerebellar peduncle, Heschl’s gyrus, superior temporal gyrus, and posterior limb of the internal capsule.

Jung et al. used resting-state functional connectivity MRI (rs-fcMRI) to evaluate associations of UHL with networks of executive functions in the brain [29]. Rs-fcMRI is based on the premise that the blood oxygen level-dependent (BOLD) signal in different areas of the brain varies temporally in sync if they are functionally connected [30]. They hypothesized that children with UHL have different patterns of functional connectivity responsible for auditory and executive functions, thus explaining the behavioral and educational difficulties documented among these children. Indeed, they noted that there were several areas with adaptive (i.e. strengthened connections between networks) and potentially maladaptive functional connections (i.e. weakened connections and lack of suppression of default mode networks) in executive function networks.

Although the studies include small numbers of children with UHL, with potential for significant bias due to selection and participation bias, and unknown correlation versus causation, they suggest intriguing and troubling associations of UHL with cognitive development. What is still unknown is whether congenital or early-onset UHL can affect educational attainment, choice of work/employment/profession, and participation in society as adults.

## Balance and Vestibular Function

Based on the observation that children with bilateral HL frequently have problems with balance and vestibular function, Wolter et al. hypothesized that UHL could also affect balance in children [31]. They performed a case-control study of 14 children with UHL (mean age 15.8 years), 9 with cochlear nerve deficiency, compared with 14 children with NH (mean age 16.1 years) on a validated clinical test of balance, the Bruininks-Oseretsky Test-2 (BOT-2), and time to fall. The BOT-2 scores were significantly worse in children with UHL, and time to fall was significantly worse only in the most difficult tasks when visual and somatosensory inputs were limited. They interpreted these results to suggest that not only auditory but also vestibular function may be affected in children with UHL. Indeed, given the high rate of temporal bone anomalies in children with UHL, it should not be surprising that they might have a high rate of vestibular dysfunction and difficulty with balance.

## Quality of Life

Quality of life (QOL) has been defined as “individuals’ perception of their position in life in the context of the culture and value systems in which they live and in relation to their goals, expectations, standards, and concerns” [32]. In addition to the functional auditory deficits, HL can have implications for a child’s self-perceived well-being in physical, emotional, social, and mental domains. Many generic QOL assessments have been used for children with HL but lack sensitivity to change with intervention and specificity to hearing loss. To date, the only hearing-related QOL measure that has been validated in children with UHL is the Hearing Environments and Reflections of Quality of Life (HEAR-QL) [33, 34]. In both the 7–12-year-old (HEAR-QL-26 for children) and 13–18-year-old (HEAR-QL-28 for Adolescents) versions, hearing-related QOL was significantly decreased in children with UHL compared to children with NH, but highly variable. Children and adolescents with UHL had HEAR-QL scores that were not statistically different from children and adolescents with bilateral HL. For adolescents, the HEAR-QL scores were influenced negatively not only by the presence of any hearing loss but also by female sex, lower level of maternal education, and use of hearing devices.

In a systematic review and meta-analysis of QOL in children with HL, Roland et al. showed that UHL affects the social and school domains of children’s QOL as measured by the PedsQL [35••]. Children with UHL had lower scores (worse QOL) than those with NH in the school domain by 8.79 points (95% CI 4.03–13.55), and the social domain by 4.31 points (95% CI 0.26–9.22). These differences are statistically and clinical meaningful. Additionally, a statistically significant difference was seen in total score for children with UHL (lower by 3.8 points, 95% CI 0.2–7.4); however, this difference did not meet the threshold for clinical significance. Clinically significant differences were considered those with an absolute value 4, as previous studies have identified a minimal clinically important difference of 4 to 6 points for the PedsQL.

## Interventions in Children with UHL

Two recent systematic reviews have summarized studies of hearing rehabilitation for children with UHL. Appachi et al. evaluated auditory outcomes from various modalities of hearing rehabilitation, including FM systems, conventional hearing aids, CROS hearing aids, and bone-conduction hearing devices [36••]. FM systems were found to be beneficial for speech recognition in noise, conventional hearing aids demonstrated trends toward improvement in speech perception, and CROS aids had mixed auditory outcomes. Bone-conduction aids showed consistent gain in speech reception thresholds (SRT) and speech discrimination, improvement in hearing in noise, but inconsistent results with sound localization. There was also documentation of improved QOL functional outcomes as measured by the CHILD. Liu et al. also performed a systematic review evaluating the role of bone conduction aids [37]. They, too, found consistent gain in SRT and speech discrimination, but inconsistent results with sound localization. However, the measurement of QOL shows high rate of usage and benefit in the learning domain.

Early published studies of cochlear implants (CI) in children with UHL now document at least 36 cases, whose age at CI varied from 10 months to 11 years [11, 38–41]. The indications for CI were mixed, from congenital to short-duration post-lingual profound UHL. The outcomes reported include improvement in SRT, CNC words, AZBio sentences in quiet, and sound localization. In addition, most reported consistent use of CI as a marker for acceptance and benefit.

Despite the current enthusiasm for fitting hearing amplification devices and early trials for cochlear implantation in children with UHL, assessment of outcomes after these interventions is still in its infancy. Although there is widespread hope that improving binaural stimulation and restoring cortical integration of bilateral acoustic information will have a positive effect on the developmental outcomes of children with UHL, we do not have firm evidence to make those claims as yet.

## Conclusions and Unknowns

UHL in children has been demonstrated to negatively impact speech-language development, cognition, and quality of life. However, many clinicians can readily identify children with UHL who do not seem affected by these consequences, and it is likely that some adapt well to hearing in only one ear. Thus, learning which predictors put some children with UHL at greater risk for delays is crucial to recommending management. Although some studies suggest early intervention might be helpful in mitigating the detrimental effects of UHL, more rigorous investigation with greater numbers of children over time is needed to establish its effectiveness. Increasingly widespread acceptance of amplification for children with UHL should be accompanied by studies that confirm beneficial outcomes. In addition to amplification, parents need education, and coaching to enrich the child’s language environment and incidental learning at home. Our professional challenge is to demonstrate that any of these interventions improve the outcomes important to the child and family.

What is still unknown:

- How can tests of binaural function, such as sound localization and binaural summation, be performed and normed in young children?
- If amplification is recommended, at what age should it begin? This is predicated on the question of the auditory critical period for children with UHL, and knowing whether the benefits of early intervention are worth the possible costs.
- Should children with profound UHL receive CI? This question is predicated on whether the benefit of CI is worth the risk of surgery and the costs of this device. In addition, if CI is recommended for children with UHL, what is the maximum duration of deafness that should be allowed?
- What do we recommend for children with profound UHL and cochlear nerve aplasia or hypoplasia? Should they receive some type of amplification or be allowed to compensate for themselves?
- Do children with UHL have permanent decrease in language and cognition (disability) or do they have slower acquisition (delay)?
